# Quantified ischemic core’s radiological hypodensity and risk of parenchymal hematoma in > 4.5 h-window stroke thrombectomy

**DOI:** 10.1038/s41598-020-73280-0

**Published:** 2020-10-01

**Authors:** Alba Chavarría-Miranda, Bárbara Yugueros, Beatriz Gómez-Vicente, Miguel Schüller, Jorge Galván, Miguel Castaño, Ana I. Calleja, Elisa Cortijo, Mercedes de Lera, Javier Reyes, María Begoña Coco-Martín, Jesús Agulla, Mario Martínez-Galdámez, Juan F. Arenillas

**Affiliations:** 1 Stroke Program, Department of Neurology, Hospital Clínico Universitario, Valladolid, Spain; 2 Clinical Neurosciences Research Group, Department of Medicine, University of Valladolid, Valladolid, Spain; 3 Neuroradiology Unit, Department of Radiology, Hospital Clínico Universitario, Valladolid, Spain; 4 Neurovascular Research Laboratory, Instituto de Biología Y Genética Molecular, Universidad de Valladolid - Consejo Superior de Investigaciones Científicas, Madrid, Spain

**Keywords:** Neuroscience, Imaging

## Abstract

We aimed to study the relationship between the ischemic core’s (IC) radiological hypodensity and the risk of parenchymal haematoma after endovascular therapy (EVT) in acute ischemic stroke (AIS) presenting > 4.5 h from onset. We studied AIS patients with a proximal anterior circulation occlusion > 4.5 h from symptoms onset treated with primary EVT. The IC regions of interest (ROI) were manually delineated on pretreatment CT within the affected hemisphere and their specular ROIs on the unaffected side. IC hypodensity ratio was calculated by dividing mean Hounsfield Unit (HU) value from all ROIs in affected/unaffected hemisphere. Primary endpoint: parenchymal hematoma (PH) type hemorrhagic transformation. Secondary: poor long-term clinical outcome. From May 2015 to November 2018, 648 consecutive AIS patients received reperfusion therapies and 107 met all inclusion criteria. PH after EVT was diagnosed in 33 (31%) patients. In bivariate analyses, IC hypodensity ratio (*p* < 0.001) and minimum HU value (*p* = 0.008) were associated with PH. A lower IC hypodensity ratio [OR < 0.001 (< 0.001–0.116) *p* 0.016] predicted PH but not poor clinical outcome in multivariable logistic regression models. A lower IC radiological density predicted a higher risk of PH in > 4.5 h-window AIS patients treated with primary EVT, although it was not independently associated with a worse clinical outcome.

## Introduction

Endovascular therapy (EVT) is now considered the standard of care in acute ischemic stroke (AIS) from proximal arterial occlusion in the cerebral anterior circulation. Moreover, after the positive result of the DWI or CTP Assessment with Clinical Mismatch in the Triage of Wake-up and Late Presenting Strokes Undergoing Neurointervention with Trevo (DAWN) trial^1^ and Endovascular Therapy Following Imaging Evaluation for Ischemic Stroke 3 (DEFUSE 3)-trials^2^, the time window for EVT has been broadened up to 24 h, as already included in updated guidelines^3^. As described in those trials´ methodology, selection of patients for EVT beyond 6 h after symptoms onset is based on the detection of a favorable mismatch profile between the extent of infarct core and the volume of salvageable brain tissue on advanced brain imaging. However, as time elapses within this delayed window, the necrotic process affecting the infarct core may evolve, as reflected by a progressively lower radiological density. Accordingly, in these delayed patients the persistent ischemic penumbra can be surrounding a markedly hypodense ischemic core, which often elicits the question whether it is too dark to be safely treated among the members of the stroke team.

In line with this, the prognostic significance of the degree of infarct core’s radiological hypoattenuation in the setting of EVT-driven cerebral reperfusion is largely unknown. In the intravenous thrombolysis literature, both the extent of early infarction signs and the degree of hypoattenuation on baseline computed tomography (CT) scan, were associated with a higher risk of symptomatic hemorrhagic transformation and unfavorable long-term clinical outcome in acute ischemic stroke patients receiving intravenous t-PA^4–6^. Nevertheless, late-window patients treated with EVT in the DAWN and DEFUSE-3 trials did not receive intravenous t-PA, and therefore it is not clear whether the degree of infarct core’s radiological hypodensity has an impact on the risk of hemorrhagic complications after primary endovascular cerebral reperfusion.

With this background, we aimed to investigate the relationship between the ischemic core’s degree of radiological hypodensity on admission’s brain CT and the risk of hemorrhagic transformation after > 4.5 h-window endovascular therapy for anterior circulation AIS. As a secondary objective, we studied the association of infarct’s core hypodensity degree with long-term functional outcome.

## Methods

We designed a retrospective, observational, unicentric study based on a prospective registry of all consecutive AIS patients treated with reperfusion therapies at our tertiary Stroke Center between May 2015 and November 2018. The study was approved by the Clinical Investigation Ethic Committee (CEIC) of the Hospital Clínico Universitario of Valladolid. Treatment of personal, clinical and radiological data obtained in the registry was done in accordance with the Spanish law of Personal Data Protection. Informed consent was obtained from all subjects or, if subjects under 18, from a parent and/or legal guardian. The database as well as the CT images are available for other researchers upon reasonable request.

### Patient selection

Patients were eligible if they fulfilled the following criteria: (1) Time from symptom onset to presentation at hospital > 4.5 h, including wake up strokes and unknown beginning; (2) Absence of previous relevant disability evaluated by the modified Rankin Scale score (mRs) (pre-stroke mRs score < 2); (3) Patients with an AIS affecting proximal anterior cerebral circulation considered elegible for EVT. Selection criteria for endovascular thrombectomy included substantial neurological deficit and confirmed intracranial large vessel occlusion affecting either intracranial internal carotid artery (ICA) or the first segment of middle cerebral artery (MCA), with a target mismatch profile on CT perfusion, following DEFUSE criteria^7^. Exclusion criteria for EVT included intracranial hemorrhage at baseline CT or extent early ischemic signs defined by an Alberta Stroke Program Early CT Score (ASPECTS) score < 4. The degree of ischemic core’s hypodensity based on initial visual inspection of CT images is not considered an exclusion criterion for EVT in our protocol. (4) No intravenous thrombolysis prior to thrombectomy; (5) No past history of ischemic or hemorrhagic stroke.

### Clinical protocol

Clinical management of all patients before, during and after endovascular therapy was performed in accordance with our Institutional Protocol, which is based on updated national and international guidelines and includes strict post-thrombectomy blood pressure control^3^. Clinical variables included in our Reperfusion Registry comprise sex, age, presence of hypertension, diabetes, dyslipemia, smoking or alcohol consumption, previous treatment with antiplatelets or anticoagulants, baseline stroke severity evaluated by NIHSS scale, last seen normal time, systolic and diastolic initial blood pressure, and routine blood work-up data like platelet count and glycemia level prior to reperfusion treatment. Patients were treated under general anesthesia or conscious sedation using a femoral access and it was also registered. We also gathered information about time from onset to door, door to first neuroimaging, door-groin puncture time, and time from groin puncture to last angiographic series. Whenever possible patients were admitted to our Stroke Unit after completing the endovascular treatment; If general anesthesia with orotracheal intubation was needed during the procedure, they first temporarily went to the Reanimation or Intensive Care Unit. Stroke subtypes were categorized as atherothrombotic, cardioembolic, other causes or due to an undetermined etiology^8^.

### Neuroimaging protocol

#### Admission imaging

Acute neuroimaging was performed upon admission and included non-contrast CT, CT-angiogram, and CT perfusion. All CT imaging was carried out using a General Electric Lightspeed CT (Waukeshad, WI, USA), equipped with 64 rows of detectors. Perfusion source images were automatically processed online using a MIStar software server (Apollo Medical Imaging Technology, Melbourne, Australia) in order to assess the presence of target mismatch to allow EVT indication. Target mismatch was defined by a core volume of less than 70 cc and a penumbra: core ratio greater than 1.8. A reduction in cerebral blood flow < 30% compared to the contralateral side was used for core volume definition in CTP images. The extent of early ischemic changes was assessed using ASPECTS scale on pretreatment non-contrast CT, and the site of arterial occlusion was visualized on the CT-angiogram. Reperfusion status was assessed on the final cerebral angiogram series and graded according the modified Thrombolysis in Cerebral Infarction (mTICI) scale. Successful reperfusion was defined as TICI grade 2b or 3.

#### Core processing

Quantification of the ischemic core’s degree of hypodensity was performed by a team of two investigators (BYB, ACM) with extensive training in stroke CT interpretation. Offline imaging processing was conducted using Horos Software for iMac. Infarct core ROIs were traced on plain CT images attending early signs of ischemia, due to the limited coverage of the used CT perfusion scanner that precludes a complete assessment of the ischemic core in the majority of the cases. Given that the image evaluators were blind to all clinical information, CT perfusion images were used only as a guide to initially localize the ischemic brain tissue. Early ischemic signs considered to demarcate the infarct core included effacement or hypodensity of cortical-subcortical structures, loss of differentiation between gray and white matter and swallowing of cortical sulci. Those affected areas were manually traced as regions of interest (ROI) in the affected hemisphere, and symmetrical ROIs were generated in the unaffected hemisphere. Cerebrospinal fluid and chronic ischemic areas were carefully avoided when defining ROIs limits. In absence of visible early ischemic changes on plain CT, ROIs were arbitrarily placed on ASPECTS M1 regions of both hemispheres, including cortical and subcortical brain tissue. Both investigators defined ROIs limits individually and a consensus read was done to obtain definite ROIs limits that were used for further analysis. In case no consensus was reached, a third read by a stroke neurologist with expertise in brain imaging (JFA) was performed. In order to remain blinded to outcome data, baseline CT images were anonymized and a numeric code was used. The imaging software calculated the radiological density of the designed ROIs using Hounsfield Units (HU), and the mean and minimal HU values for each ROI were registered. This procedure was repeated for each CT slice were core ROIs appeared. The following parameters were used for statistical analysis: minimum HU obtained within the infarct core; mean HU observed in all selected ROIs in the affected and unaffected sides, and core hypodensity ratio, calculated as the quotient between the mean HU value obtained from the ROIs of the affected hemisphere and the mean HU value obtained in the specular ROIs of the unaffected side.

#### Evaluation of hemorrhagic transformation

A new brain CT was performed after 24 h of treatment or earlier in case clinical deterioration occurred. Different hemorrhagic transformation subtypes were categorized according to the European Cooperative Acute Stroke Study (ECASS-2)^6^ definition in hemorrhagic infarction (HI) type 1 and 2, and parenchymal hematoma type 1 and 2^5^. Symptomatic hemorrhage was defined as hemorrhagic transformation that was associated with significant neurological worsening, as defined by an increase in four or more points in the NIHSS scale within the first 24 h (ECASS 3)^9^. A new brain CT was repeated at 48–72 h to differentiate between hemorrhagic transformation and radiologic contrast retention when the 24-h CT scan was considered doubtful in this respect.

### Primary safety and efficacy endpoints

The predefined safety endpoint was the presence of parenchymal haematoma type hemorrhagic transformation, defined as parenchymal haematoma types 1 (PH1) or 2 (PH2) on control brain imaging. The efficacy endpoint was long-term clinical outcome, assessed by the score obtained at three months in the modified Rankin Scale score, with a score 0–2 considered indicative of functional independence. Preferably, Rankin scale was rated by a stroke neurologist during a physical visit performed at our stroke prevention clinic, or by telephone if the patient belonged to remote referring areas. Symptomatic hemorrhagic transformation was considered a secondary safety endpoint.

### Statistical analysis

Statistical analyses were carried out using SPSS statistics version 24 (Chicago, Illinois, USA). Baseline continuous variables were described using their mean ± standard deviation or median (interquartile range), as appropriated. Discrete variables were expressed as number of cases and their percentage. Kolmogorov–Smirnov test was used to evaluate if the variables followed a normal distribution. Bivariate analyses were performed to detect baseline variables associated with the occurrence of the primary and secondary endpoints. Comparison between variables was performed using the Chi-square test for discrete variables, t-student test for quantitative variables with a normal distribution and Mann–Whitney U test for quantitative variables not following a normal distribution. Those variables related to radiological hypodensity were analyzed as continuous variables and also categorized into variable tertiles. After that, we performed multivariate adjusted regression models to identify baseline predictors of the different endpoints. The ischemic core’s hypodensity parameter with the highest statistical significance in bivariate analyses was included into the models, and adjustment was performed by all variables showing a *p* < 0.1 in the respective bivariate models. Data are presented as adjusted odds ratios (OR) and respective 95% confidence intervals (CI). Finally, Receiver Operating Characteristic curves (ROC) were conducted to determine the best discriminatory ROC cut off points of the significant radiological hypodensity variables. Statistical significance was defined as a *p* value < 0.05.

## Results

From May 2015 to November 2018, 648 AIS patients were treated with reperfusion therapies at our Stroke Center, 243 of whom presented beyond > 4.5 h from LSN (last seen normal) time. From those 243 patients treated, 117 presented MCA-M1 or terminal ICA occlusions and were treated with primary EVT. Twelve of these 117 patients were excluded because their CT did not have sufficient quality for core delineation due to movement artifacts, and the final number of included patients was 105. The mean age of the study group was 73.4 ± 11.5 years, women proportion was 45% and median baseline NIHSS score was 16 (interquartile range 10–20). The main demographic, clinical and radiological baseline variables appear in Table 1. The median time from LSN to door was 490 min (interquartile range 327–690).

**Table 1:** Clinical, demographic and radiologic characteristics of the study population (n = 105).

Age	73.4 ± 11.5
Sex (female)	48 (44.9%)
Smoking (current or past)	20 (18.7%)
Alcohol consumption	10 (9.3%)
Hypertension	74 (69.2%)
Diabetes	28 (26.2%)
Dyslipemia	36 (33.6%)
Antiplatelets	25 (23.4%)
Anticoagulation	24 (22.4%)
Baseline NIHSS	16 (10–20)
Known time of symptom onset	73 (68.2%)
Pretreatment blood glucose level	130.3 ± 33.8
Admission systolic blood pressure	146.8 ± 23.9
Admission dyastolic blood pressure	78.2 ± 13.2
Platelets	208,845.3 ± 82,217.4
**TOAST classification**	
Cardioembolic	54 (50.5%)
Atherothrombotic	19 (17.8%)
Undetermined	30 (28.0%)
Other	4 (3.7%)
Onset-to-door time	490 (327–690)
Door-to-puncture time	97 (78–113)
Door-to-last angiographic serie time	40 (25–75)
Conscious sedation anesthesia	69 (64.5%)
Baseline ASPECTS	8 (7–9)
M1 Oclussion	87 (81.3%)
**Hemorrhagic transformation**	
Parenchymal hematoma (PH1)	25 (23.4%)
Parenchymal hematoma (PH2)	8 (7.4%)
Symptomatic hemorrhagic transformation	9 (8.6%)
Complete recanalization (TICI 2b-3)	92 (86.0%)
Mean HU at affected hemisphere	29.5 ± 3.6
Mean HU at non-affected hemisphere	32.6 ± 2.8
Minimum core HU	28.5 ± 4.1
Ischemic core’s hypodensity ratio	0.89 (0.85–0.94)

NIHSS, National Institute of Health Stroke Scale; TOAST, Trial of Org 10,172 in acute stroke registry; TC, Computerized Tomography; ASPECTS, Alberta Stroke Program Early CT Score; M1, medium cerebral artery segment M1; PH1, parenchymal hematoma type 1; PH2, parenchymal hematoma type 2; TICI, Thrombolysis in Cerebral Infarction; HU, Hounsfield Units.

**Figure 1:**
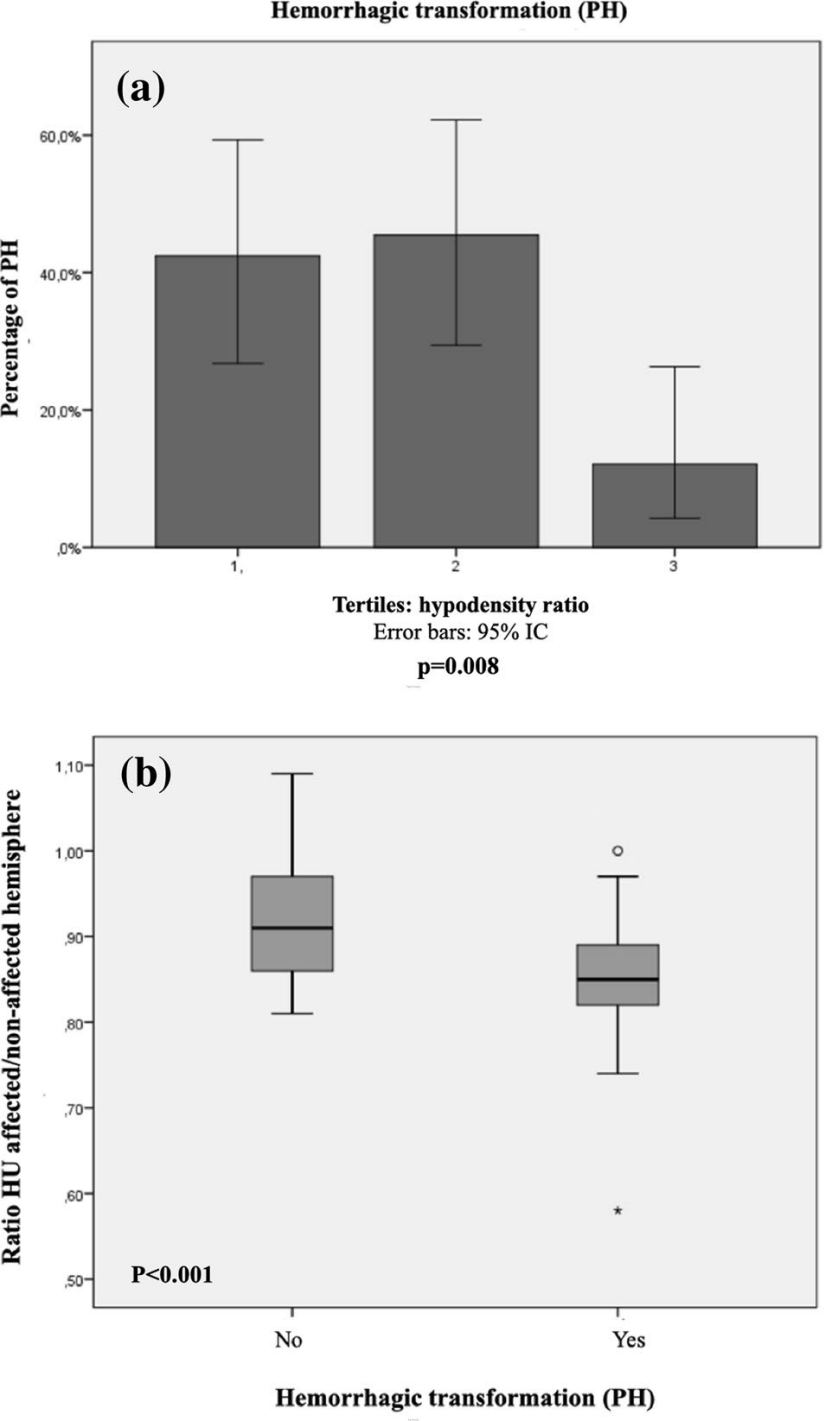
Ischemic core’s radiological density and PH-type hemorrhagic transformation. (**a**) Barr chart: percentage of patients with PH-type hemorrhagic transformation by hypodensity ratio tertiles. Error bars: 95% CI. Tertile 1: 0.58–0.86. Tertile 2: 0.87–0.92, tertile 3: 0.93–1. *p* = 0.027. (**b**) Box-plot showing the relationship between hypodensity ratio and PH type hemorrhagic transformation. Horizontal superior line, percentile 75; inferior horizontal line, percentile 25.

Parenchymal hematoma was diagnosed in 33 patients (31%), 25 (23.4%) were PH1 and 8 (7.4%) PH2. Two patients did not have CT after endovascular reperfusion therapy. Table 2 shows the results of bivariate analysis between baseline variables and the occurrence of PH. Significant associations were found for all parameters related to quantified ischemic core hypodensity: mean HU, (*p* = 0.018), minimum core HU value (*p* = 0.008) and ischemic core hypodensity ratio (*p* < 0.001). In all cases, the lower the core’s radiological density, the higher the risk of developing a PH. The rest of variables associated with hemorrhagic transformation type PH are shown on Table 2. When the hypodensity variables were categorized into tertiles, significant associations were found between the core’s hypodensity ratio and PH risk (*p* = 0.027) and between the minimum core’s HU value and PH risk (*p* = 0.008), as shown on Fig. 1, with increasing PH risk observed with lower hypodensity tertiles. The result of multivariate adjusted logistic regression model is shown on Table 3. The core hypodensity ratio emerged as an independent predictor of PH [OR < 0.001 (< 0.001–0.116) *p* = 0.016], in the sense that the lower the hypodensity ratio (i.e. the darker the brain tissue), the higher the risk of developing a PH. As shown on Table 3, additional PH predictors were a lower ASPECTS score, incomplete cerebral reperfusion and general anesthesia. We then generated a ROC curve using the variables core hypodensity ratio and PH transformation. Although the area under the curve (AUC) was significant (AUC 0.73, *p* < 0.001), we could not find an optimal cutoff value with enough sensitivity and specificity so as to dichotomize the variable of interest.

**Table 2:** Bivariate analysis between baseline outcomes and hemorrhagic transformation PH type.

Outcome	No PH (n = 72)	Yes PH (n = 33)	*P* value
Age	73.1 ± 11.8	73.7 ± 11.3	0.81
Sex (female)	35 (48.6%)	12 (36.4%)	0.24
Smoking (current or past)	14 (19.4%)	5 (15.2%)	0.60
Alcohol consumption	5 (6.9%)	4 (12.1%)	0.38
Hypertension	51 (70.8%)	21 (63.6%)	0.46
Diabetes	17 (23.6%)	10 (30.3%)	0.47
Dyslipemia	22 (30.6%)	12 (36.4%)	0.56
Antiplatelets	16 (22.2%)	8 (24.2%)	0.82
Anticoagulation	13 (18.1%)	9 (27.3%)	0.28
Baseline NIHSS	15 (9–19)	19 (15–22)	0.012
Known time of symptom onset	51 (70.8%)	20 (60.6%)	0.30
Pretreatment blood glucose level	130.13 ± 34.36	130.41 ± 33.50	0.97
Admission systolic blood pressure	144.5 ± 23.9	152.2 ± 23.9	0.13
Admission dyastolic blood pressure	77.5 ± 13.5	80.1 ± 12.3	0.35
Cardioembolic stroke	33 (45.8%)	19 (57.6%)	0.44
Onset-to-door-time	463 (323.5–688.3)	655 (374.5–740.0)	0.17
Door-to-CT-time	22 (15.5–34.8)	22(15.0–37.5)	0.77
Door-to-puncture time	98 (79.3–114.8)	94 (74.5–110.5)	0.67
Groin puncture-to last angiographic serie time	40 (25–60)	35 (17.5–87.5)	0.74
General anesthesia	54 (75%)	15 (45.5%)	0.003
Baseline ASPECTS	9 (8–10)	7 (6–8)	< 0.001
M1 Oclussion	58 (80.6%)	28 (84.8%)	0.60
Complete recanalization (TICI 2b-3)	66 (93%)	26 (78.8%)	0.04
Mean HU at affected hemisphere	30.1 ± 3.7	28.3 ± 3.0	0.018
Mean HU at non-affected hemisphere	32.6 ± 3.0	32.7 ± 2.1	0.94
Minimum HU	29.2 ± 4.1	26.9 ± 3.7	0.008
Ischemic core’s hypodensity ratio	0.91 (0.86–0.97)	0.85 (0.82–0.89)	< 0.001

Continuous data are expressed as mean ± standard deviation or as median and interquartile range. Categorical data are expressed as number of cases (n) and its percentage. *P* value < 0.05 was considered significant.

**Table 3:** Logistic regression: hemorrhagic transformation PH type.

Variable	OR (95% CI)	*P* value
Baseline ASPECTS	0.713 (0.519–0.979)	0.037
Complete recanalization (TICI 2b-3)	0.153 (0.031–0.749)	0.021
General anesthesia	4.397 (1.543–12.531)	0.006
Ischemic core’s hypodensity ratio	< 0.001 (< 0.001–0.116)	0.016

Only those variables that emerge as independent predictors of PH are shown.

OR, odds ratio; CI, confidence interval.

Symptomatic hemorrhagic transformation was diagnosed in nine patients (8.6%). No significant associations were found between the different hypodensity variables and the risk of symptomatic hemorrhagic transformation neither on bivariate analysis, nor in logistic regression (data not shown). Regarding long-term clinical outcome, a lower infarct core’s mean HU (*p* = 0.04) and a lower minimum HU (*p* = 0.05) were significantly associated with a worse functional prognosis three months after stroke in the bivariate analysis. Two patients could not be evaluated at 90 day follow up. Result of this analysis is shown on Table 4. However, quantified hypodensity was not independently associated with long-term outcome when the multivariate logistic regression model was applied.

**Table 4:** Bivariate analysis between baseline variables and long term functional prognosis.

Variable	Bad outcome (n = 63)	Good outcome (n = 40)	*P* value
Age	75.7 ± 10.9	69.6 ± 12.0	0.009
Sex (female)	31 (49.2%)	17 (42.5%)	0.51
Smoking (current or past)	6 (9.5%)	14 (35.0%)	0.001
Alcohol consumption	5 (7.9%)	4 (10.0%)	0.72
Hypertension	47 (74.6%)	24 (60.0%)	0.12
Diabetes	20 (31.7%)	7 (17.5%)	0.11
Dyslipemia	22 (34.9%)	13 (32.5%)	0.80
Antiplatelets	16 (25.4%)	8 (20.0%)	0.53
Anticoagulation	15 (23.8%)	8 (20.0%)	0.65
Baseline NIHSS	19 (14–22)	10 (8–16)	< 0.001
Alcohol consumption	42 (66.7%)	29 (72.5%)	0.53
Pretreatment blood glucose level	138.05 ± 35.07	117.64 ± 25.39	0.002
Admission systolic blood pressure	148.3 ± 22.3	141.3 ± 25.0	0.15
Admission dyastolic blood pressure	79.4 ± 14.0	75.6 ± 11.9	0.16
Cardioembolic stroke	34 (54.0%)	18 (45.0%)	0.59
Onset-to-door time	569.0 (325.0–685.0)	543.0 (324.0–698.3)	0.76
Door-to-CT time	22.0 (15.0–40.0)	21 (17.3–25.8)	0.44
Door-to-puncture time	98.0 (75.0–119.0)	95.5 (75.8–106.5)	0.49
Groin puncture-to-last angiographic serie time	45.0 (27.0–98.0)	32.5 (20.0–52.8)	0.04
General anesthesia	30 (47.6%)	6 (15%)	0.001
Baseline ASPECTS	8 (7–9)	9 (7–10)	0.13
M1 oclussion	50 (79.4%)	34 (85.0%)	0.47
Complete recanalization (TICI 2b-3)	50 (79.4%)	38 (97.4%)	0.01
Mean HU at affected hemisphere	28.8 ± 3.1	30.3 ± 4.0	0.04
Mean HU at non-affected hemisphere	32.2 ± 2.5	33.1 ± 3.0	0.08
Minimum HU	27.7 ± 3.7	29.4 ± 4.6	0.05
Ischemic core’s hypodensity ratio	0.88 (0.85–0.93)	0.90 (0.86–0.96)	0.12

Continuous data are expressed as mean ± standard deviation or as mean and interquartile range. Categorical data are expressed as number of cases (n) and its percentage. *P* value < 0.05 was considered significant.

## Discussion

The main finding of this observational study, performed in anterior circulation AIS patients treated with primary mechanical thrombectomy beyond 4.5 h from symptoms onset, is that the quantified degree of ischemic core’s radiological hypodensity emerged as a predictor of parenchymal hematoma after endovascular reperfusion. The darker the ischemic core’s brain tissue appeared on pretreatment plain CT, the higher the risk of developing a type PH hemorrhagic transformation after EVT. Our finding may be clinically relevant, given the reported relationship between PH and futile reperfusion after endovascular therapy^10^. Thus, late-window thrombectomy patients with more hypodense ischemic cores on CT might obtain a lower benefit from EVT given their higher risk of substantial hemorrhagic transformation. However, we failed to encounter an independent association between the degree of hypodensity and long-term clinical outcome, although this negative finding could be due to an insufficient sample size. Therefore, our results do not allow the identification of a subgroup of late-window AIS patients who may not benefit from EVT.

The main methodological novelty of our study is that we focused mainly on the quantification of the ischemic core’s degree of darkness, and not only assessed its extent or volume, as most previous studies in the field of hemorrhagic risk after EVT had done. In this regard, our results are complementary of prior reports showing that the extent of the ischemic core, assessed either using ASPECTS score or the quantified core volume, is an independent predictor of hemorrhagic transformation after reperfusion therapies^11,12^. Of note, the rate of hemorrhagic transformation PH-2 in our study group was slightly higher than the one reported by the HERMES group (7.4 vs 5.1), which raises the question whether this could be due to the patients being treated in a wider time window^13^. Remarkably, in our study ischemic core’s hypodensity ratio predicted PH risk independently of ASPECTS score, which implies that even small dark-evolved ischemic cores might be more prone to bleeding after EVT. Moreover, following international guidelines, we did not treat patients with large cores. There is still uncertainty as whether patients with large cores may benefit from EVT. The second HERMES metanalysis provided preliminary data suggesting that carefully selected patients with large cores (ASPECTS 3–5) may have more favorable functional outcomes despite an increased risk of ICH^14^. In line with these results, the SELECT trial^15^ showed that in patients with unfavorable CT/favorable CTP, thrombectomy was associated with a reduction in mortality compared to medical management with similar safety outcomes regarding sICH. There are clinical trials underway to clarify this issue, in which after our results, the darkness of the ischemic core could be assessed together with core volume to evaluate its relative contribution to bleeding risk in this particular subset of EVT patients.

As a potential pathophysiological explanation for the relationship observed between brain tissue hypodensity and PH risk, we hypothesize that a lower radiological density may indicate a more evolved necrotic process within the ischemic core, with a more pronounced damage to neurovascular unit and the blood–brain barrier^16^. In line with this hypothesis, previous studies have found a good correlation between lower Hounsfield Units measured in areas with early ischemic changes on CT, and higher intensity of those areas on FLAIR sequences on Magnetic Resonance Imaging^17^. This implies that most of our AIS patients with dark ischemic cores might have had clearly hyperintense lesions on MRI-FLAIR imaging, which would have meant an exclusion criterion for MRI-based late-window thrombolysis clinical trials such as the WAKE-UP study^18^. Another additional hypothesis could be that late-window patients may have a more evolved postischemic inflammatory response, with molecules involved in blood brain barrier disruption being more expressed at late window in those areas showing a more profound damage to the neurovascular unit^19^. If this hypothesis was true, these > 4.5 h-window patients might especially benefit from neuroprotective therapies administered as an add-on to endovascular therapy, as suggested by some authors^20^.

Other independent predictors of PH in our study were low ASPECTS score, absence of complete reperfusion and EVT performed under general anesthesia. The first two have been previously reported in the literature^11,12,21^. Regarding general anesthesia, our results are not in line with the findings reported in randomized clinical trials for general anesthesia vs. conscious sedation, where general anesthesia was not associated with an increased intracranial bleeding risk^22–24^ In contrast, other studies have found opposite results like ours^25^, so the relationship between EVT outcome and anesthesia modality is far from being clarified. Given its observational nature, our study was not designed to evaluate the impact of anesthesia on late-window patients treated with EVT. Our results may be influenced by the fact that general anesthesia at our Institution is reserved for clinically unstable or uncooperative patients, or when technical difficulties are foreseen. Further studies are needed to ascertain whether general anesthesia may be associated with a particular risk in late-window patients.

Our study has some limitations. First, we have studied a selected and homogeneous sample of AIS patients with anterior circulation proximal arterial occlusions presenting beyond 4.5 h from onset treated with primary EVT, so our results may not be generalizable to < 4.5 h patients and to those patients with prior intravenous thrombolysis. Second, the sample size was limited, so the study may be underpowered to detect significant differences in long-term outcome. Third, ROI demarcation was based on visual inspection of CT imaging and manual delineation in several steps, which may lead to interobserver variability and limit the clinical value of our algorithm. Automated methods of radiological density processing with infarct core delineation based on whole-brain CTP images, may be the optimal way to evaluate the clinical importance of this parameter. Fourth, postprocedural blood pressure values were not included in our prospective reperfusion registry and could not be considered in the statistical analysis. Elevated blood pressure (BP) values after mechanical thrombectomy have been identified as predictors of post-procedural intracranial haemorrhage, worse long-term prognosis and higher mortality^26^, so we cannot rule out an influence of BP control on hemorrhagic transformation risk in our series, although BP was carefully managed according to our protocol in all patients post EVT. Finally, the clinical applicability of this result is limited by our failure to obtain a good cutoff hypodensity value in the ROC curve able to discriminate high-risk patients.

In conclusion, a lower ischemic core’s radiological density predicted a higher risk of PH-type hemorrhagic transformation in extended window AIS patients treated with primary endovascular therapy, although it was not independently associated with a worse long-term clinical outcome. This finding deserves to be replicated in prospective and larger studies. We suggest that the variable core hypodensity ratio could be analyzed in future clinical trials and prospective registries in the field of EVT.
